# Circular RNA CDK6 suppresses cervical cancer proliferation and metastasis by sponging miR-449a

**DOI:** 10.1080/21655979.2022.2036898

**Published:** 2022-02-13

**Authors:** Peilin Zhong, Aihua Guo, Linhua Wang, Xiurong Lin, Mei Feng

**Affiliations:** Department of Gynecology, Fujian Medical University Cancer Hospital, Fujian Cancer Hospital, Fuzhou, Fujian, China

**Keywords:** Circular RNA CDK6, cervical cancer, cell proliferation, miR-449a, EMT

## Abstract

Cervical cancer is a malignant tumor that severely threatens female health. Recently, more and more studies indicated that circRNA could function as a tumor activator or suppressor in cervical cell development. Therefore, we aimed to study the effect of circRNA CDK6 (circCDK6) on the development and biological behavior of cervical cancer. We used quantitative real-time PCR (qRT-PCR) to examine the circCDK6 expression level in cervical cancer cell lines. RNA-fluorescence in situ hybridization confirmed the location of circCDK6 and miR-449a in HeLa and CaSki cells, respectively. Then, the biological function of silencing circCDK6 in cellular proliferation, metastasis, and Epithelial-Mesenchymal Transition (EMT)-related process was determined. We also performed RNA Binding Protein Immunoprecipitation (RIP) and Dual-luciferase reporter assay to determine the relationship between the circCDK6 and miR-449a. Finally, the results showed that circCDK6 level remarkably increased in several cervical cancer cells, especially in Hela and CaSki cells. The miR-449a was further confirmed to be a potential target of circCDK6, and its expression increased by silencing circCDK6. The circCDK6 participated in tumorigenesis and cancer progression and might serve as a tumor suppressive factor in cervical cell progression via Epithelial-MesenchymalTransition (EMT) process by regulating miR-449a.

## Graphical Abstract

**Figure:**
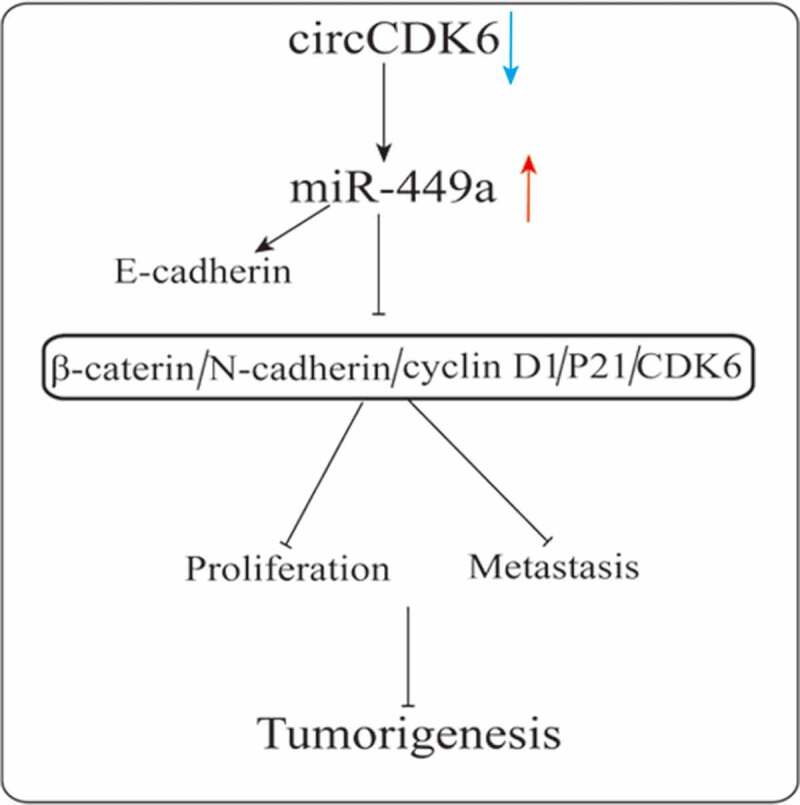

## Introduction

Cervical cancer is a malignant tumor that severely threatens female health. It causes about 265,700 deaths worldwide every year [1,2]. Although different strategies have been adopted for cervical cancer therapy, such as surgery, radiotherapy, and chemotherapy, the cancer still poses a serious threat to women’s health [3]. Pathological studies confirmed that the molecular mechanism of cervical cancer growth and metastasis is very complex; it involves many gene mutations and epigenetic changes [4–6]. Therefore, further elucidation of the internal mechanism of cervical cancer growth and metastasis is of great value for cervical cancer treatment.

Circular RNA (circRNA) is a non-protein coding RNA with covalent closed loop; it is unaffected by the RNA exonuclease [7,8]. circRNA expression is more stable and less degradable due to its characterization [9]. Recently, circRNAs were found to have a critical effect on various cellular processes. Studies show that circRNA could be a tumor activator or suppressor in cervical cell development. The main mechanism of circRNAs is miRNA sponging, that is, circRNAs act as a kind of scaffold to interact with proteins to exert their biological effects [10–13]. For example, has_circRNA_101996 mediated miR-8075 and regulated TPX2, thus contributing to cell growth and metastasis of cervical cancer [11]. Circ-LARP1B contributed to cutaneous squamous cell carcinoma progression by targeting miR-515-5p/TPX2 axis [14]. Therefore, these evidences indicated the significance of the transcriptional regulation of circRNAs and provided novel insights into the progression of cervical cancer.

MicroRNAs (miRNAs) are conserved; linear noncoding RNAs are 19–25 nt in length [15,16]. miRNAs generally have a regulatory effect on gene expression by coming in contact with the 3’-UTR of mRNA [17], thus inhibiting protein translation or mRNA degradation [17]. The aberrant expression level of miRNA was involved in various types of malignant tumors; it participated in many cellular functions [18,19]. Some miRNAs are involved in cervical cancer occurrence and development. The miR-449a, located on chromosome 5q11.2 is a possible target in several cancer cell lines, such as breast cancer cells [20], T-cell lymphoma cells [21], and gastric cancer [22]. The purpose of our study was to explore the cellular roles of circCDK6 and its underlying mechanism in cervical cancer cells.

Therefore, to examine the influences of circCDK6 on the developmental and biological behavior of cervical cancer, we investigated circCDK6 expression level in cervical cancer cell lines. We detected the biological effect of silencing circCDK6 on cellular proliferation, metastasis, and Epithelial-Mesenchymal Transition (EMT)-related process. We also performed RNA Binding Protein Immunoprecipitation (RIP) and Dual-luciferase reporter assay to determine the relationship between circCDK6 and miR-449a. We aimed to elucidate the mechanism of circCDK6 in cervical cancer at the cellular level and to provide a new theoretical basis for the diagnosis and treatment of cervical cancer.

## Materials and methods

### Cell culture

The cells (Hela, SiHa, C33A, and CaSki) used in the study were from ATCC (catalog number: CRM-CCL-2, HTB-35, HTB-31 and CRL-1550), and HaCat was acquired from BeNa Culture Collection(BNCC339817, China). DMEM (Dmem medium) medium containing 10% FBS (GIBCO, NY) was used for cell culture in the incubator at 37°C with 5% CO_2_. The cells (CaSki and HeLa) in the logarithmic growth stage were digested, centrifuged, and resuspended every 2 to 3 days. The cells were cultured on the 6-well plate with 5 × 10^5^ in each well. When they grew to a density of about 60%–70%, they were ready for transient transformation. Based on the instruction of Lipofectamine 2000 kit, si-circCDK6 (50 pmol) and miR-449a inhibitor (200 nM) were used for transfection.

### qRT-PCR assay

As described by Chen, et al [23]. TRIzol reagent was utilized for extracting RNA from cells. The cDNA of circ CDK6 and mRNA was acquired by PrimeScript RT Kit. Quantification of circCDK6, miR-449a, and miRNA was performed, and their relative levels were controlled to GAPDH (glyceraldehyde phosphate dehydrogenase gene) and U6, respectively. The primer sequences are presented in Table 1.

**Table 1.** The primer sequences

CircRNA CDK6	FORWARD	CCAAGTTTTAGCAGAAAACCTCTCC
	REVERSE	CTCCTCGAAGCGAAGTCCTC
GAPDH	FORWARD	ATGGGGAAGGTGAAGGTCG
	REVERSE	TTACTCCTTGGAGGCCATGTG
linear mRNA	FORWARD	CAGTGACTTTCCTCTGACATGC
	REVERSE	GGCTCTGCATAAAACGGCAC
GAPDH	FORWARD	ATGGGGAAGGTGAAGGTCG
	REVERSE	TTACTCCTTGGAGGCCATGTG
si-CircRNA CDK6-Homo1	Sense:	5’- AAGUUUUAGCAGAAAACCUCUTT
	Antisense:	5’-AGAGGUUUUCUGCUAAAACUUTT
si-CircRNA CDK6-Homo1	Sense:	5’- CCAAGUUUUAGCAGAAAACCUTT
	Antisense:	5’-AGGUUUUCUGCUAAAACUUGGTT
si-CircRNA CDK6-Homo1	Sense:	5’- CAGAAAACCUCUCCGCGCGAATT
	Antisense:	5’- UUCGCGCGGAGAGGUUUUCUGTT
Negtive control	Sense:	5’-AUGUUUAAAUUGUUGUGAAATT
	Antisense:	5’-UUUCACAACAAUUUAAACAUTT
miR-449a	FORWARD	CGCGTGGCAGTGTATTGTTA
miR-449a	REVERSE	AGTGCAGGGTCCGAGGTATT
miR-449a	RT Primer	GTCGTATCCAGTGCAGGGTCCGAGGTATTCGCACTGGATACGACACCAGC
U6	FORWARD	CTCGCTTCGGCAGCACATATACT
U6	REVERSE	ACGCTTCACGAATTTGCGTGTC
U6	RT Primer	AAAATATGGAACGCTTCACGAATTTG

### RNA-Fluorescence in situ hybridization

As described by Chen,et al [23]. Transfected HeLa and CaSki cells were seeded on the cover glass. Then, 4% polyoxymethelene (POM) was fixed at 4°C for 10 min, Cells in CSK buffer supplemented with 10 mm VRC and 0.5% Triton X-100 were exposed to 4°C for 10–12 min; 70% alcohol was used for 10 min, and incubation was performed at 4°C for 10 min. At −20°C, three concentrations of alcohol (70%, 85%, and 100%) were used for 5 min for dehydration. Then, the cells were air dried. We used neutral gum to stick the cell slide onto the slide (face up). The hybrid buffer (cy3-circRNA CDK6, DIG-miR-449a) was denatured at 76°C for 10 min. The 5 μl hybrid mixture was dropped on the slide, covered with a cover glass, sealed with rubber, and placed in a wet dark box at 37°C for overnight hybridization. The rubber and cover glass were subsequently discarded after hybridization. The 50% formamide was used to wash the climbing slides at 42°C for 5 min for three times. Then, the slides were washed with 2× SSC at 42°C for 5 min for three times. The DAPI reagent was utilized for nucleus straining. The locations of circCDK6 and miR-449a were captured by fluorescence microscope (Olympus FV1000).

### Actinomycin D and RNase R treatment

As described by Chen, et al [23]. Actinomycin D (2 mg/ml; Sigma-Aldrich) was used to prevent RNA transcription. RNase R (3 U/μg, Epicenter Technologies, Madison, WI, USA) was added to total RNA (2 μg) and incubated at 37°C for 30 min. After incubation with actinomycin D or RNase R, the relative expression levels of the circular and linear forms were examined. The primer sequences are presented in Table 1.

### CCK-8 assay

As described by Song, et al [11]. Transfected 1 × 10^3^ cells were seeded, and every group contained three duplicate wells. A total of 100 μl CCK-8 reagent (10%, Sigma, MO, USA) was injected into each well for different times (0, 24, 48, and 72 h). Culturing was continued for 12 h. The blank control was the culture medium without cells. The absorbance of each well was measured at the optical density (OD) of 450 nm by micro-plate reader (Bio-Rad, USA), and the inhibition rate was examined.

### Colony formation assay

As described by Song, et al [11]. Hela or CaSki cells at a density of 1 × 10^3^ were cultured onto a 6-well plate. After transfection, colonies were subsequently fixed using methanol and stained. The colonies were counted and photographed.

### Cell migration and invasion assay

As described by Song, et al [11]. Hela or CaSki cells were examined by a transwell chambers without Matrigel-coated membrane (BD Biosciences). For the invasion, the Matrigel was coated with DMEM medium and cultured on the upper chamber wells with a Matrigel. The transfected Hela or CaSki cells were digested and suspended with serum-free DMEM. Then, cells were added to the upper chamber at a density of 5 × 10^4^. The lower chamber had normal DMEM medium. After 24 h, existing cells were discarded by using cotton swabs. The migrating or invasive cells in the lower chambers were fixed, washed with PBS, and immediately stained. The stained cells were captured by an Olympus BX51 microscope (Olympus).

### RIP

As described by Zhang, et al [24]. Cells were utilized and lysed by Magna RIP RNA-binding protein immunoprecipitation Kit as previously described. The negative control was IgG.

### Dual-luciferase reporter system

As described by Song, et al [11]. Wild-type circCDK6, mutant circCDK6, and miR-449a were synthesized by Sangon Biotech (Shanghai, China) and then subcloned into psiCHECK^TM^-2. Hela or CaSki cells with a density of 3 × 10^4^ pre well were seeded in a 24-well plate. Then, we co-transfected miR-449a mimics or miR-NC with psiCHECK^TM^-2-wt-circCDK6 or psiCHECK^TM^-2-mut-circCDK6. After 48 h, the luciferase activities were examined.

### Flow cytometry

As described by Song, et al [11]. Propidium iodide (PI) staining was performed in the flow cytometry by ACSVerse (BD, USA). After 48 h, transfected Hela or CaSki cells were collected, washed, and centrifuged. The cells with a density of 5 × 10^5^ in a total volume of 200 μl cells were subsequently washed with PBS and centrifuged twice. Then, we added precooled 70% ethanol and fixed the cells overnight at 4°C. Cells were subsequently suspended in 500 μl 1× Binding Buffer, washed with PBS, and centrifuged once. Finally, 500 μl 1× Binding Buffer and 50 μl PI were added into the tube, and incubation was performed for 30 min. Treated cells were measured via flow cytometry and analyzed by ModFit. FL2-w and FL2-A were used, and the conjoined cells were removed.

### Protein extraction and western blot assay

As described by Chen, et al [23]. The treated Hela or CaSki cells were harvested and lysed by a radio immunoprecipitation (RIPA) lysis buffer (Beyotime, China). Each sample with the same amount (40 μg) was loaded on an SDS-PAGE (10%–12%), and later immediately transferred onto a PVDF membrane. Subsequently, it was blocked and incubated with the first antibodies (1:1000) at 4°C for 12 h. In another day, the treated-membrane was incubated with the appropriate HRP-labeled secondary antibodies (1:5000; Abcam) for 1 h more. Subsequently, protein expression was visualized by the ECL kit. The first antibodies utilized in the study were anti-CDK6, anti-cyclin D1, anti-N-cadherin, anti-P21 and anti-E-cadherin antibodies (1:1000; Abcam), and actin (1:3000, Abcam).

### Statistical analysis

All the results in triplicate from independent experiments were examined by the SPSS software (SPSS, Chicago) and presented as means ± standard deviation. The calculation method was examined through the Student’s t-test. p < 0.05 was statistically significant.

## Results

To examined the influences of circCDK6 on the developmental and biological behavior of cervical cancer, we used qRT-PCR to examine the circCDK6 expression level in cervical cancer cell lines. RNA-fluorescence in situ hybridization was used to confirm the location of circCDK6 and miR-449a in HeLa and CaSki cells, respectively. Then, the biological function of circCDK6 silencing in cellular proliferation, metastasis, and EMT-related process was detected. We also performed RIP and Dual-luciferase reporter assay to determine the relationship between circCDK6 and miR-449a. The results are as follows.

### circCDK6 exhibited an increased expression in cervical cancer cells

**Figure 1.**
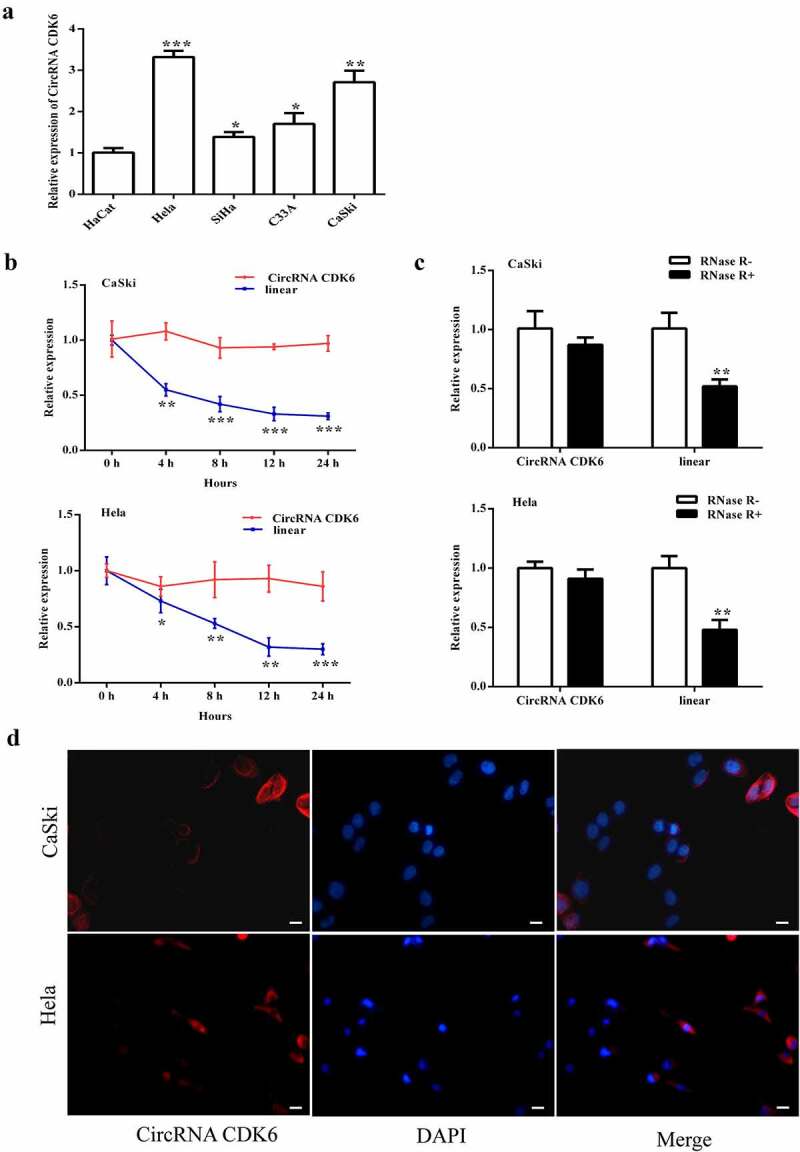
**circCDK6 is upregulated in the four cervical cancer cell lines**. A. the circCDK6 expression showed a significant upregulation in the cervical cells. B. the transcript half-life of circCDK6 in CaSki and Hela cells treated with transcription inhibitor Actinomycin D. C. RNase R was administrated to the extracted RNA to measure the relative expression of circular form (circCDK6) and its linear form in CaSki and Hela cells. D. the subcellular distribution of circCDK6 was detected using CaSki and Hela cells. The circCDK6 and the nucleus showed as red blue, respectively. The scale bar is 100 μm. *p < 0.05, **p < 0.01 and ***p < 0.001 vs. respective control.

Relative circCDK6 expression was detected in several cervical cancer cells. It showed an increase expression in four cancer cells (HeLa, SiHa, C33A, and CaSki) compared with HaCat cells. Therefore, HeLa and CaSki cells were chosen for the following experiment. Actinomycin D was used to inhibit RNA transcription. The transcript half-life of circCDK6 was notably longer than that of the linear form, suggesting its stability (Figure 1b). The extracted RNA was further added to RNase R, and the circular form became more resistant than the linear form (Figure 1c). Furthermore, the circCDK6 was positioned in the cellular cytoplasm (Figure 1d).

### Silencing circCDK6 repressed biological function in HeLa and CaSki cells

**Figure 2.**
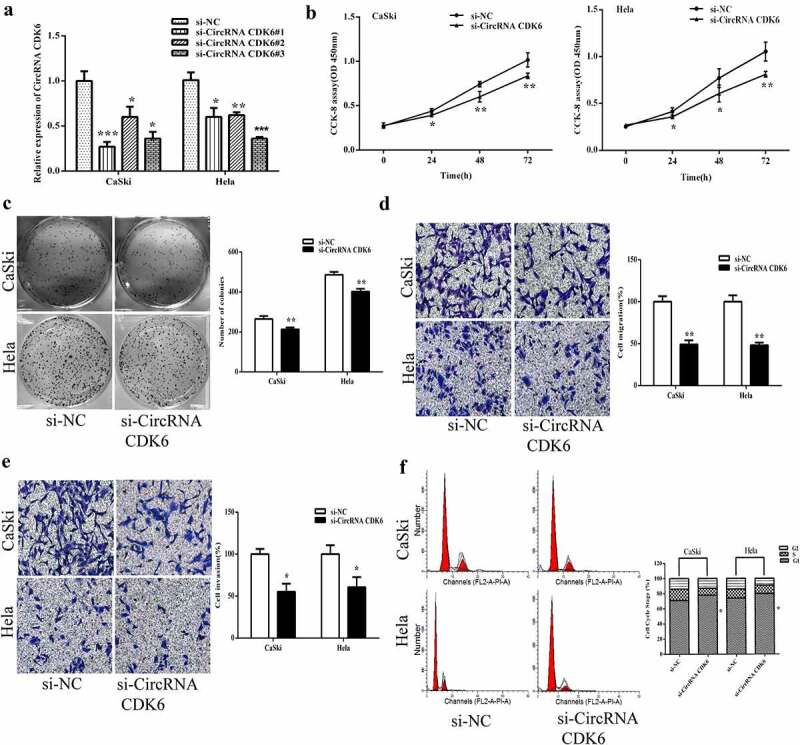
**Silencing circCDK6 influences the function of cervical cancer cells**. A. circCDK6 expression level was remarkably decreased in HeLa and CaSki cells transfected with si-circCDK6 as compared with the si-NC control. B. The proliferation in HeLa and CaSki cells transfected with si-circCDK6 at 0, 24, 48 and 72 was examined by the CCK-8 assay. C. The colony formation in the HeLa and CaSki cells transfected with si-circCDK6 was detected by the colony formation. D and E. The migration and invasion in HeLa and CaSki cells transfected with si-circCDK6 was performed by the transwell assay. F. Cell cycle in HeLa and CaSki cells transfected with si-circCDK6 was carried on the Flow cytometry. *p < 0.05, **p < 0.01 and ***p < 0.001 vs. respective control.

To assess the cellular influence of circCDK6 on cervical cancer cells, three stable silencing siRNAs against circCDK6 were transfected. As shown in Figure 2a, the two silencing siRNAs resulted in the lowest expression of circCDK6 and were chosen for the next assays. The CCK8 and colony assays showed that silencing circCDK6 markedly inhibited the proliferation of the two cell lines (Figures 2B and 2C). In addition, transwell assay indicated that silencing circCDK6 repressed the migrative and invasive ability of cervical cancer cells (Figures 2D and 2E). Flow cytometry indicated that Hela and CaSki cells were in the G1 phase after transfection with silencing shRNA against circCDK6 (figure 2f).

### circCDK6 modulates EMT relative process

**Figure 3.**
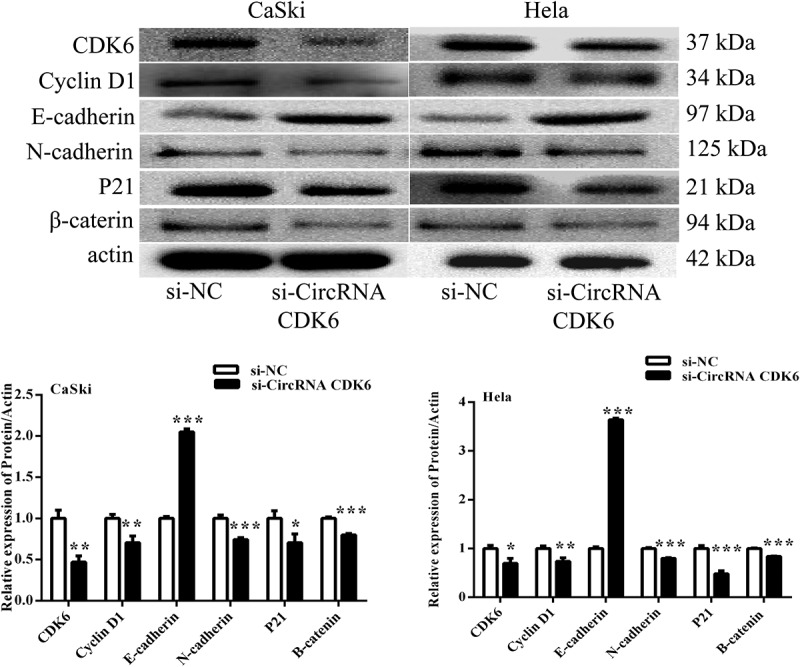
**Silencing circCDK6 modulated EMT process of cervical cancer cells**. The expression of EMT-related proteins was analysis in (a) HeLa and (b) CaSki cells transfected with si-circCDK6 by Western blotting. The sample size was 40 μg/lane. *p < 0.05, **p < 0.01 and ***p < 0.001 vs. respective control.

To explore the underlying mechanism, the influences of circCDK6 on EMT process were examined. Figure 3 shows that silencing circCDK6 suppressed the expression levels of CDK6, P21, cyclin D1, N-cadherin, and β-catenin and enhanced the relative levels of E-cadherin in HeLa and CaSki cells. Our finding suggested that circCDK6 could suppressed cervical cell metastatic behavior.

### circCDK6 exerts as a sponge of miR-449a

**Figure 4.**
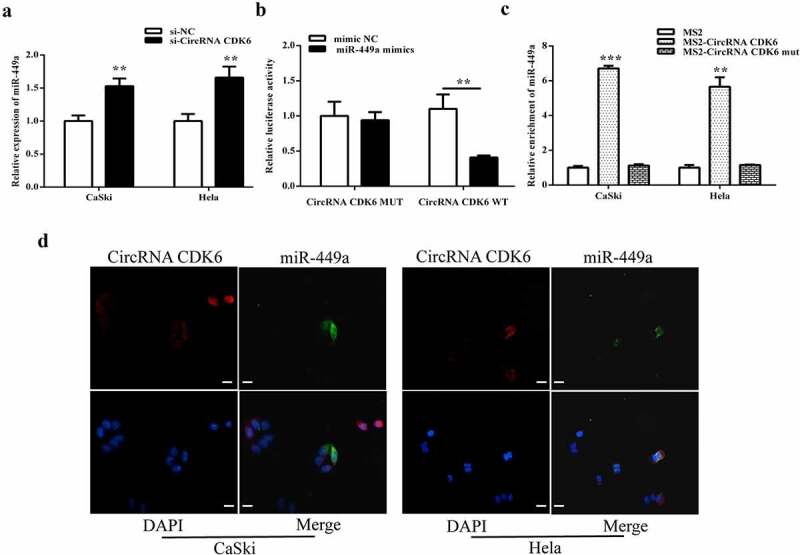
**circCDK6 exerts as the target of miR-449a in cervical cancer cells**. A. The miR-449a level in Hela and CaSki cells with transfection of circCDK6 siRNA. B. The luciferase activity in the cotransfection of miR-449a and circCDK6 wild-type or mutant. C. The relative enrichment of miR-449a in transfection of circCDK6 wild-type or mutant. D. The subcellular location of circCDK6 and miR-449a. The scale bar is 100 μm. **p < 0.01. respective control.

The miR-449a expression levels in HeLa and CaSki cells transfection by si-circCDK6 were examined. Downregulated circCDK6 markedly enhanced miR-449a expression (Figure 4a). Luciferase reporter assay revealed that cotransfection with miR-449a and circCDK6 wild-type suppressed the luciferase activity, suggesting an interaction between miR-449a and circCDK6 (Figure 4b). Moreover, RIP assay indicated that miR-449a could be pulled down by the biotinylated-circCDK6 probe (Figure 4c). Both circCDK6 and miR-449a were positioned in cellular cytoplasm (Figure 4d). All these results indicated that miR-449a can possibly be a target for circCDK6 in cervical cancer cells.

### Downregulated miR-449a alleviated the influences of silencing circCDK6 on cellular functions

**Figure 5.**
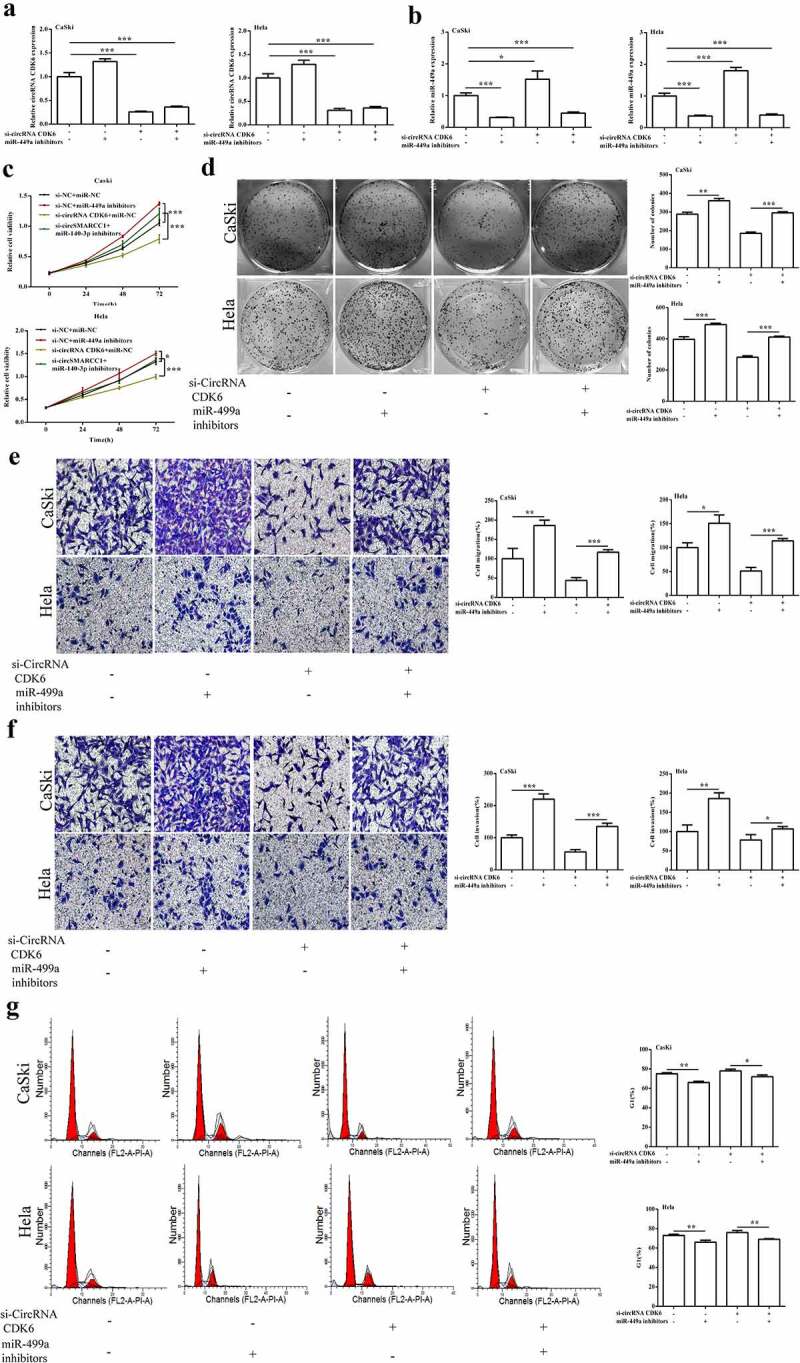
**miR-449a engaged in the influences of silencing circCDK6 in HeLa and CaSki cells**. The expression level of miR-449a (a) and circRNA CDK6 (b) in HeLa and CaSki cells transfected with si-circCDK6 and miR-449a inhibitor detected by qRT-PCR, the sample size was 1 μg/well. Influence of circCDK6 and miR-449a on cell viability assayed by CCK-8, the sample size was 1 × 10^3^/well(c), cell proliferation examined by colony formation assay, the sample size was 1 × 10^3^/well (d), migration (e) and invasion (f) detected by transwell assay(the sample size was 5 × 10^4^/well), and cell cycle detected by Flow cytometry(the sample size was 1 × 10^6^/well). *p < 0.05, **p < 0.01 and ***p < 0.001 vs. respective control.

As shown in Figure 5a and B, downregulation of miR-449a notably suppressed the increase of miR-449a expression caused by the silencing of circCDK6. However, the miR-449a inhibitor could not enhance the decrease of circCDK6 expression in Hela and CaSki cells caused by the silencing of circCDK6. Additionally, miR-449a alleviated the decrease in proliferation (Figure 5c), colony formation (Figure 5d), and metastasis (Figure 5e and F) caused by the silencing of circCDK6. Hela and CaSki cell cycles were influenced by both miR-449a and circCDK6 (Figure 5g). These above findings illustrated that miR-449a was a downstream factor of circCDK6 and regulated the effects of circCDK6 silencing on cervical cancer cells.

### miR-449a inhibitor transfection weakened the influence of silencing circCDK6 on cervical cancer cell EMT process

**Figure 6.**
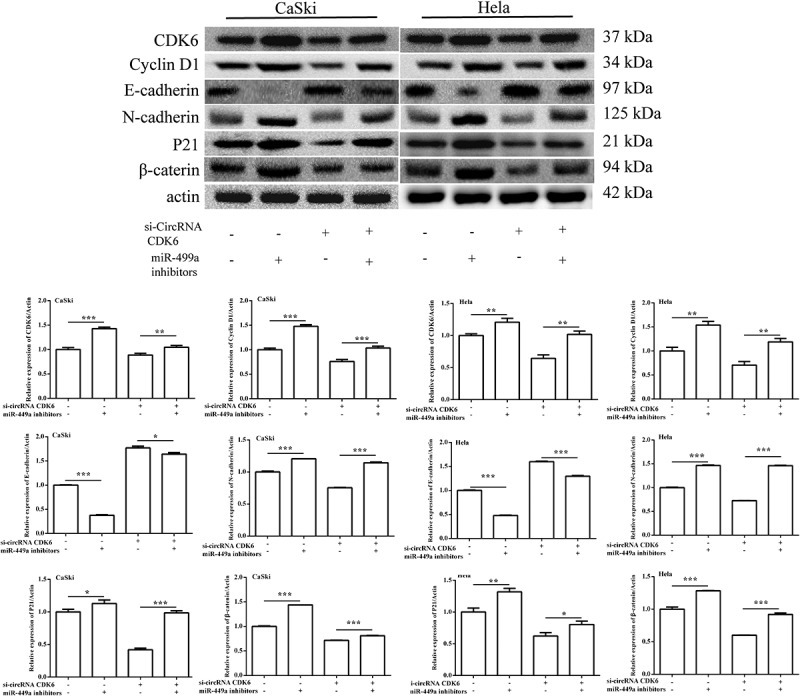
**The protein levels of the EMT-related process were examined by Western blotting**. The expression of CDK6, cyclin D1, E-cadherin, N-cadherin, P21 and β-catenin in (a) HeLa and (b) CaSki cells transfected with si-circCDK6 and miR-449a inhibitor. The sample size was 40 μg/lane. *p < 0.05, **p < 0.01 and ***p < 0.001 vs. respective control.

To determine whether miR-449a participated in the effects of silencing circCDK6 on Hela and CaSki cell EMT process, the related protein expression was examined. Figure 6 data indicated that the relative levels of N-cadherin, β-catenin, P21, and cyclin were remarkably upregulated in HeLa and CaSki after transfection by silencing circCDK6 and miR-449a compared with when transfection was conducted with si-circCDK6 alone. On the contrary, the levels of E-cadherin decreased compared with when transfection was performed only with silencing circCDK6 cells. All these data revealed that miR-449a was involved in the effects of circCDK6 silencing on the EMT process.

## Discussion

The biological roles of circRNAs in various biological activities, particularly in cancer occurrence and progression, have been attracting an increasing amount of attention in recent years. In this study, we investigated whether circCDK6 expression level increased in several cervical cancer cells (HeLa, SiHa, C33A, and CaSki). Silencing circCDK6 suppressed the growth and metastasis and inhibited EMT-related process. In addition, the relationship between circCDK6 and miR-449a was verified. Silencing circCDK6 promoted the level of miR-449a. Our data indicated that miR-449a would be a possible target to circCDK6, and miR-449a inhibitor was involved in the effects of silencing circCDK6 on Hela and CaSki cell growth, metastasis, and EMT-related process.

Previous studies have suggested that more than 90% genes are involved in non-coding RNAs [25]. As a type of critical non-coding RNAs in mammalian cells, circRNA has been previously verified to be involved in the modulation of many cell biological processes [26]. To date, several circRNAs were found to be involved in cervical cancer. For example, circRNA SMARCA5 expression was upregulated, influenced the proliferation rate, and positively correlated with ERK1 and ERK2 levels [27]. circRNA HIPK3 targeted miR-338-3p and enhanced cellular EMT [28]. CircRNA CLK3 was remarkably upregulated, and it modulated cellular process [14]. In the study, circCDK6 was first discovered and showed a high expression level in cervical cancer cells. After silencing circCDK6, proliferation was decreased, which indicated that circCDK6 was a critical factor in cervical cancer cell growth.

Tumor metastasis is a highly complex process and is the primary cause of poor prognosis of cervical cancer. Here, silencing circCDK6 remarkably repressed cancer migration and invasion. The EMT-related biological process is the lineage transition between epithelium and mesenchyme, that is, the tumor cells lose their adhesion and are involved in embryogenesis and diseases, i.e., tumor migration and invasion [29]. E-cadherin and N-cadherin are two important factors that are involved in the EMT process. E-cadherin is an epithelial cell adhesion factor [30], and its decrease could result in the loss of cellular junction. The increase of N-cadherin is involved in the EMT process [31,32]. These two factors have been described in various epithelial cancers and are associated with malignancy and metastasis. In the study, silencing circCDK6 inhibited cancer cell metastasis through the decrease of N-cadherin and the increase of E-cadherin. Findings exhibited that circCDK6 was involved in the modulation of cervical cancer metastasis.

Many circRNAs serve regulatory functions by modulating the expression of miRNAs. As a tumor suppressive factor, miR-449a reportedly showed decreased expression levels in both cervical cancer tissues and cells [33]. miR-449a would be a possible target of circCDK6. miR-449a inhibitor notably alleviated the influence of silencing circCDK6 on cell proliferation, metastasis, and EMT-related process. This finding suggested that miR-449a is a downstream factor of circCDK6 and that the influence of circCDK6 on cervical cancer proliferation and metastasis might also be achieved by regulating miR-449a.

## Conclusion

In conclusion, circCDK6 was found to be a tumor suppressive factor in cervical cancer proliferation and metastasis. Silencing circCDK6 inhibited cell proliferation, cell migration, and invasion by increasing miR-449a expression and by modulating E-cadherin and N-cadherin expressions. These findings might provide a comprehensive insight into the function of circRNA in the regulatory network of cervical cancer for its diagnosis.

## Data Availability

All data are in this article.

## Highlights

1. The regulatory role of circCDK6 and miR-449a was discussed for the first time;
2. The mechanism of action of circCDK6 on cervical cancer was clarified for the first time;
3. circCDK6 participates in tumorigenesis and cancer progression and might be a tumour suppressive factor in cervical cell progression via EMT process by regulating miR-449a.
